# The supercritical CO_2_ process does not affect the mechanical properties and the microarchitecture of trabecular bone at the microscopic scale: A microindentation and microcomputed tomography study

**DOI:** 10.1016/j.bonr.2025.101859

**Published:** 2025-07-25

**Authors:** Théo Krieger, Virginie Taillebot, Aurélien Maurel-Pantel, Claire Camy, Grégoire Edorh, Matthieu Ollivier, Martine Pithioux

**Affiliations:** a Aix Marseille Univ, CNRS, ISM, 13009 Marseille, France; b Aix Marseille Univ, APHM, CNRS, ISM, Sainte-Marguerite Hospital, Institute for Locomotion, Department of Orthopaedics and Traumatology, 13009 Marseille, France; c BIOBank®, Tissue Bank, 77127 Lieusaint, France; d Ecole Centrale Marseille, 13013 Marseille, France; e Aix-Marseille University, CNRS, Centrale Marseille, LMA, Marseille, France

**Keywords:** Bone allograft, Trabecular bone, Supercritical CO_2_, Irradiation, Microarchitecture, Microindentation

## Abstract

Bone allografts are frequently used in many surgical procedures because of their osteoconductive and osteoinductive properties. After being extracted from the donor, the graft is treated with a process that cleans and sterilizes it before being implanted in the patient. While they guarantee the safety of the patient receiving the graft, preservation processes often affect bone properties.

This study aims to measure the effect of a supercritical CO_2_ process on the microarchitecture and the mechanical properties of trabecular bone at a microscopic scale using microindentation. 7 femoral heads were harvested from patients who had undergone a total hip arthroplasty. 42 cubic samples of 10 mm side from these femoral heads were randomly distributed in 3 groups: frozen at −20 °C, gamma irradiated and frozen at −20 °C, and treated with a supercritical CO_2_ process including gamma irradiation. All samples were imaged by microtomography and characterized by microindentation to correlate the bone microarchitecture with the mechanical properties at a microscopic scale.

Our results show that the supercritical CO_2_ process exerts no significant effect on the microarchitecture parameters, indentation elastic modulus, and indentation hardness.

The correlations between the microarchitecture and the mechanical properties revealed that gamma irradiation appears to induce a slight alteration in mechanical properties. However, the process combining a supercritical CO_2_ treatment and gamma irradiation does not induce any more alterations to the material than gamma irradiation itself. Thus, the supercritical CO_2_ process has no more impact than gamma irradiation on the mechanical properties of trabecular bone at the microscopic scale.

## Highlights

• The supercritical CO_2_ process does not significantly affect the microarchitecture of trabecular bone.
• The supercritical CO_2_ process does not significantly affect the indentation elastic modulus and hardness of trabecular bone.
• For a given BV/TV, supercritical CO_2_ treatment alone does not affect the indentation elastic modulus and indentation hardness.

## Graphical abstract

**Figure:**
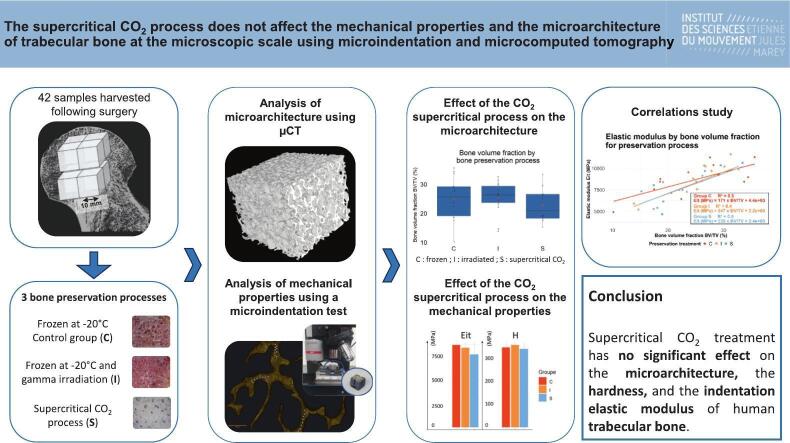

## 1 Introduction

Bone grafts are widely used by surgeons for their osteoconductive, osteoinductive, and osteogenic properties (Park et al., 2013) to support bone regeneration (Khan et al., 2005). In bone grafting, autografts are considered to be the gold standard (Sen and Miclau, 2007; Chai et al., 2011), but allografts are also widely used because they avoid donor-site morbidity (Chai et al., 2011; Oryan et al., 2014) and can provide a high-volume graft needed in big defect fractures. Trabecular bone is frequently used for bone allografts. It is a porous anisotropic composite of mostly hydroxyapatite, collagen, and water (Keaveny et al., 2001), composed of plates and rods (Gibson, 1985) oriented mainly along the main loading axes (Ketcham and Ryan, 2004). In order to guarantee the safety of the patient, sterilization and decellularization processes (Crapo et al., 2011; da Rocha et al., 2023) are used on allografts to minimize the risk of disease transmission and immune reactions before implantation in the patient (Eastlund, 1995; Delloye, 1994; Tomford, 1995). However, these processes often alter the microarchitectural and mechanical properties of the bone (Bow et al., 2019) while the microarchitecture and the mechanical properties of the trabecular bone graft must be preserved to guarantee successful bone regeneration (Boyce et al., 1999). Indeed, the conservation of both the structure and the mechanical properties of trabecular bone allows ingrowth and infiltration of fibrovascular tissue in the graft and limits the risk of postoperative fractures (Tsukamoto et al., 2022). Among the large number of preservation processes, one approach is to preserve bone marrow by cryopreservation (Salai et al., 2000) or by freeze-drying (Samsell et al., 2019). This step is often followed by gamma sterilization. Another approach is to remove bone marrow by delipidation to improve osteoconductive capacity and effectiveness of the chemical sterilization (Pruss et al., 1999) with no loss of mechanical properties (Thorén et al., 1995; Delloye et al., 2007). Among processes used to remove bone marrow, supercritical CO_2_ delipidation is widely used (Schwiedrzik et al., 2011; Mitton et al., 2005). It is known to allow viral inactivation (Fages et al., 1998) and preserve osteoconduction properties of trabecular bone (Frayssinet et al., 1998) with little effect on collagen fiber structure (Fages et al., 1994).

Although the preservation processes usually conserve the microarchitecture (Beaupied et al., 2006; Lee and Jasiuk, 2014), they often affect the mechanical properties (Vastel et al., 2004; Barth et al., 2011; Krieger et al., 2025). This has been widely studied on a macroscopic scale with tensile, bending, and compression tests (Krieger et al., 2025). These processes can also have an influence on bone properties at the microscopic scale, i.e., on the material that composes bone at the bone trabeculae scale. To investigate the microscopic scale, a microindentation test can be classically used to assess indentation hardness and indentation elastic modulus. In terms of results, it was previously shown that the preservation process using enzymatic maceration significantly reduces hardness in comparison with water maceration (Yin et al., 2010). Dehydration using ethanol increased hardness by 10 % (Dall'Ara et al., 2007) and indentation elastic modulus by 15 % (Bushby et al., 2004). Gamma sterilization appears to increase bone hardness, although not all studies agree (Allaveisi et al., 2014; El-Hansi et al., 2020; Nikolaeva et al., 2023a). However, to the best of our knowledge, no studies have yet been carried out on the impact of supercritical CO_2_ on the mechanical properties of bone at the microscopic scale.

The objective of this study was to assess the effect of a supercritical CO_2_ process on the microarchitecture and mechanical properties of trabecular bone at the microscopic scale using microindentation. X-ray microcomputed tomography (μCT) was performed to obtain a 3D volume of bone and analyze its microarchitecture. We analyzed the most commonly analyzed parameters for trabecular bone such as bone volume fraction (BV/TV), trabecular thickness (Tb.Th), trabecular separation (Tb.Sp), degree of anisotropy (DA), and connectivity density (Conn.D) (Perchalski et al., 2018; Chappard, 2010). Concerning the mechanical properties, the indentation elastic modulus and the indentation hardness were studied using microindentation. Furthermore, correlations between the mechanical and microarchitectural parameters were also studied. The effect of supercritical CO_2_ was investigated on allografts that had undergone supercritical CO_2_ preservation, including gamma irradiation. This process was compared with −20 °C frozen allografts as a reference and the gamma irradiation process alone.

## 2 Materials and methods

### 2.1 Sample preparation

For this study, 7 Femoral heads were harvested from 4 men and 3 women, aged 67 ± 15 years, who had a total hip arthroplasty. All the procedures followed the ethical standards and rules of the Aix-Marseille University Ethics Responsible Committee (N/Réf: 2021-06-03-12). From each femoral head, 6 cubes with an edge length of 10 mm, were studied. These cubes were cut with a band saw, and then distributed randomly and independently of their position in three groups corresponding to the three preservation processes studied. For each femoral head, 2 cubes were directly frozen at −20 °C (group C) (Kaye et al., 2012), 2 cubes were gamma irradiated with a dose of 28 ± 3 kGy (Nikolaeva et al., 2023a; El-Hansi et al., 2020)and then frozen at −20 °C (group I), and 2 cubes were treated by a process with a supercritical CO_2_ delipidation followed by chemical treatment and gamma irradiation with a dose of 28 ± 3 kGy (group S) and then stored in a sterile pocket at room temperature. This last process is known as the Supercrit® process (BIOBank®, Lieusaint, France) (Mitton et al., 2005). The group C represented the control group, since freezing has no significant effect on microarchitecture compared to freshly harvested bone grafts (Taillebot et al., 2024). A total of 42 samples (14 per group) were analyzed.

### 2.2 Microarchitecture analysis with X-ray microcomputed tomography

Group C and group I samples were initially thawed and rehydrated in a PBS solution (Phosphate-Buffered Saline), and group S samples were only rehydrated. All samples had the same rehydration time of 40 min and were analyzed by X-ray microcomputed tomography with the RX Solutions® EasyTom XL 150 “Mechanic Ultra” CT Scanner (Chavanod, France), with a 150 kV micro-focus RX generator tube. Intensity was set at 201 μA. The acquisition frequency was 4 fps. The imager was a 1920 × 1536 pixel^2^ Planar Matrix Sensor with a 10 μm resolution. Throughout the test, the samples were immersed in a PBS solution to avoid drying and prevent heating.

The reconstruction of the 3D images was done with RX® software. The 3D images were then processed with the ImageJ software (Schneider et al., 2012) with the BoneJ plugin (Domander et al., 2021). First, the 3D images were cropped to retain only the trabecular bone without the debris left by the cutting process during the sample preparation. The “Otsu” method (N. Otsu, 1979; Parkinson et al., 2008) was used to segment bone. Finally, BoneJ's “purify” function was applied to the 3D images to remove artifacts.

The parameters calculated were the bone volume ratio (BV/TV), trabecular thickness (Tb.Th), trabecular spacing (Tb.Sp), trabecular number (Tb.N) (Parfitt et al., 1987), degree of anisotropy (DA), trabecular volume density (Conn.D), and the main trabecular direction.

### 2.3 Analysis of mechanical properties with a microindentation test

**Fig. 1:**
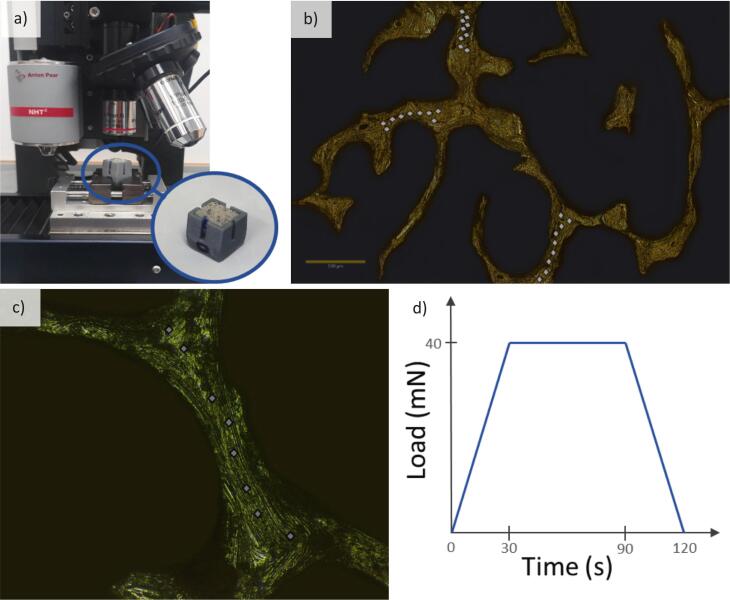
*(a) Microindentation tester with a zoom on the sample holder*. *(b) Bone section taken with the microindentation tester, on which the indents are marked with diamonds. (c) Polished surface of trabecular bone taken with the microindentation tester. (d) Microindentation loading profile.*

The bone samples were inserted in a sample holder to simplify their handling for both grinding and indentation processes (Fig. 1a). The indented face was perpendicular to the main orientation of bone trabeculae. The main orientation of bone trabeculae was found thanks to the analysis of the microarchitecture using the “Anisotropy” BoneJ's function. The indented face was polished on gradually reduced grain size discs (2500, 800 grits) under constant water irrigation and then polished with 6 μm, 3 μm, 1 μm, and 0.25 μm diamond pastes (ESCIL, Chassieu, France). Between each step, the sample was cleaned with a waterjet and then immerged in an ultrasonic bath (Elma™, Singen, Germany) for 2 min. After being polished, the samples were cleaned in the ultrasonic bath for 5 min and hydrated in a PBS solution for 12 h before indentation (Wolfram et al., 2010). To ensure proper hydration, the sample was rehydrated for 45 min between the indentation of each bone trabecula. The microindentation tester (Antonn Paar, NHT^2^, Austria) was located in a thermally controlled room at 21 °C. The indentation process followed the same protocol as previously published (Camy et al., 2024; Soldati et al., 2022; Renault et al., 2020; Roseren et al., 2022; Dall'Ara et al., 2007). Forty points were selected using a x20 microscopic objective (Fig. 1b) on five various trabeculae located on the polished surface of the sample. For each trabecula, a maximum of 3 points were placed at the intersection with other trabeculae, and a maximum of 5 points were placed along the trabecula centerline to avoid any border effect (Fig. 1c). A trapezoidal loading profile was applied (Fig. 1d) with a loading rate of 80 mN/min for 30s, a 60s pause, and an unloading rate of 80 mN/min for 30s (max load 40 mN). The indentation tests were performed with a diamond Berkovitch-type indenter, base length 120 nm (Elastic modulus: 1141 GPa; Poisson's ratio: 0.07). The trabecular bone Poisson's ratio was assumed to be 0.3 (Peters et al., 2018; Rodriguez-Florez et al., 2013). The mechanical properties measured and calculated according to the Oliver and Pharr method (Oliver and Pharr, 1992) are the indentation elastic modulus (Eit) and the indentation hardness (H). A total of 1527 indents were analyzed for an average of around 7 points kept per trabeculae.

### 2.4 Local study of the indented bone trabeculae: Correlation between mechanical parameters and thickness of indented bone trabeculae

Previous studies on the relationship between mechanical properties and bone thickness have used the average thickness throughout the sample volume (Wojtkow et al., 2019). In this work, we chose to analyze the correlation between the mechanical properties of the trabeculae and their inherent thickness.

**Fig. 2:**
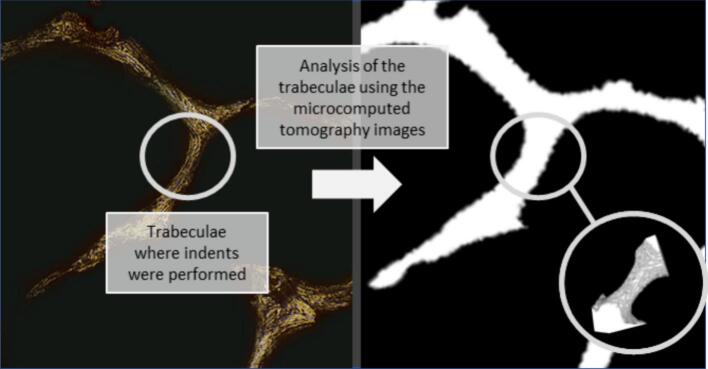
Steps of analyzing the thickness of a bone trabecula.

Each indented trabecula was identified within the 3D volume image obtained by the microcomputed tomography scanner and analyzed using ImageJ (Fig. 2). The morphology of the 5 indented trabeculae (rod or plate) of each sample was determined using the ellipsoid factor (Doube, 2015). If the ellipsoid factor was less than the chosen threshold of 0.25, the trabecula was considered as plate, otherwise as rod. Then the local thickness of each trabecula was measured using the basic measurement tools provided by ImageJ.

### 2.5 Statistical analysis

In this study, only statistical significance tests with a maximum of two variables were used. To choose the significance test to use, a Shapiro-Wilk test for normality and a Fisher test for homogeneity of variance were performed on all data. Normally distributed data with equal variance were analyzed with a parametric Student's *t*-test. The other data were analyzed with a non-parametric Mann-Whitney test.

Secondly, the coefficient of determination (R^2^) and Pearson's product-moment correlations were calculated to study the correlations between microarchitectural parameters and mechanical properties.

Finally, the Fisher-z test was used to study the effect of the preservation process on the correlation between the indented trabeculae thickness and the mechanical properties.

A result was considered statistically significant at *p* < 0.05.

## 3 Results

### 3.1 Influence of the preservation process

**Fig. 3:**
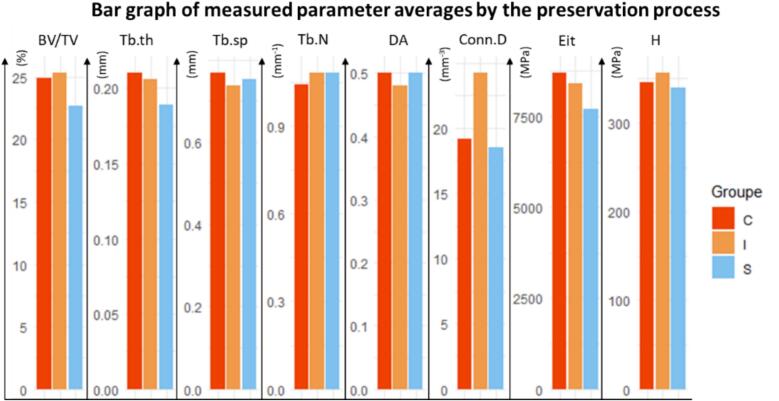
Bar graph of measured parameter averages by treatment.

Data on the effect of the preservation process on the microarchitectural parameters and mechanical properties (Eit, H) are presented in Table 1 and Fig. 3. The results show there were no significant differences between the three groups studied in either microarchitectural parameters or mechanical properties.

**Table 1:** Effect of the three processes on the microarchitectural parameters and mechanical properties on a microscopic scale.

	Frozen (control group C)	Irradiated (group I)	Supercritical CO_2_ process (group S)	p_value_ between group C and group S	p_value_ between group I and group S
Microarchitectural parameters					
BV/TV (%)	24.9 ± 7.4	25.3 ± 5.6	22.7 ± 5.6	0.39	0.23
Tb.Th (mm)	0.210 ± 0.036	0.206 ± 0.030	0.189 ± 0.026	0.09	0.12
Tb.Sp (mm)	0.767 ± 0.141	0.738 ± 0.130	0.752 ± 0.113	0.76	0.76
Tb.N (mm^−1^)	1.04 ± 0.13	1.08 ± 0.15	1.08 ± 0.13	0.47	0.97
DA	0.50 ± 0.11	0.48 ± 0.10	0.50 ± 0.11	0.86	0.60
Conn.D (mm^−3^)	19.2 ± 12.0	24.2 ± 14.0	18.5 ± 9.4	0.77	0.29

Mechanical properties					
Eit (Mpa)	8690 ± 1774	8414 ± 2064	7704 ± 1888	0.17	0.35
H (Mpa)	345 ± 56	356 ± 81	340 ± 45	0.82	0.48

### 3.2 Correlation between bone volume fraction and the mechanical properties

A correlation between indentation elastic modulus (Eit) and bone volume fraction (BV/TV) was studied using a linear regression method like that used by Renault et al. (Renault et al., 2020). Firstly, considering all preservation processes, the correlation between indentation elastic modulus and BV/TV resulted in the following equation:

$$E_{\mathrm{it}}\left(\mathrm{MPa}\right)=212\times \mathrm{BV}/\mathrm{TV}+3092\;\left(R^{2}=0.48,p_{\mathrm{value}}<0.0001\right)$$

**Fig. 4:**
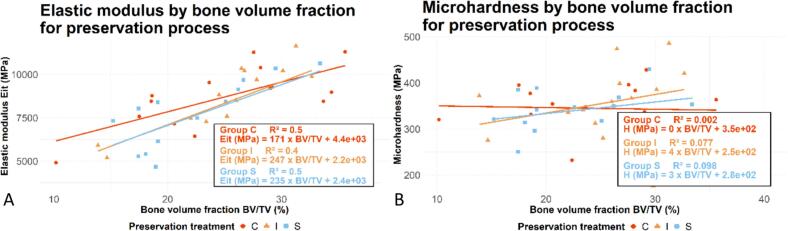
(A) Correlation curves between the indentation elastic modulus and the bone volume fraction. (B) Correlation curves between the microhardness and the bone volume fraction.

The correlation equation estimates the indentation elastic modulus as a function of the bone volume fraction. The estimation made for each preservation process independently is illustrated in Fig. 4A. For the same bone volume fraction, bones treated with gamma irradiation (I) and bones treated with supercritical CO_2_ (S) have, on average, a lower indentation elastic modulus than the frozen group (C). The slope and the intercept are similar for group I and group S, indicating that the supercritical CO_2_ process alone would have no influence on the indentation elastic modulus for a given bone volume fraction.

The same was done for estimating indentation hardness (Fig. 4B), but no correlation was found between bone volume fraction and indentation hardness (Pearson's product-moment correlation test: $p_{\mathrm{value}}=0.28$).

### 3.3 Correlation between the indentation elastic modulus and the indentation hardness

A correlation between indentation elastic modulus (Eit) and indentation hardness (H) was studied using a linear regression method (Evans et al., 1990). First, considering all preservation processes, the correlation between indentation elastic modulus (Eit) and indentation hardness (H) was calculated, resulting in the following equation:

$$E_{\mathrm{it}}\left(\mathrm{MPa}\right)=21\times H\left(\mathrm{MPa}\right)+964\;\left(R^{2}=0.46,p_{\mathrm{value}}<0.0001\right)$$

**Fig. 5:**
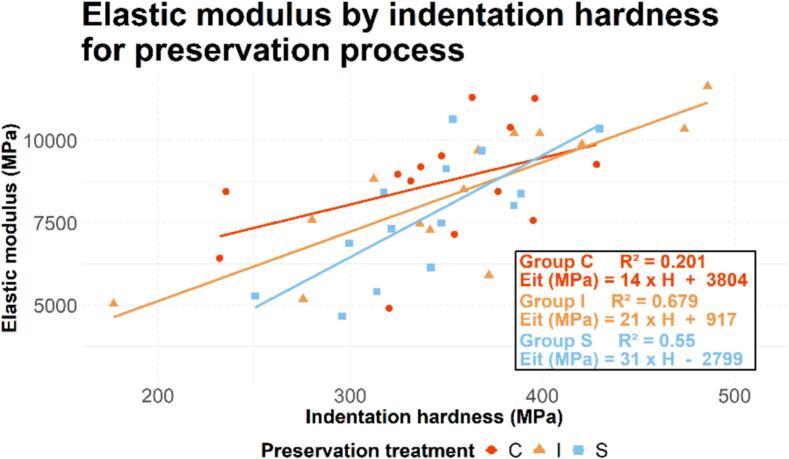
Correlation curves between the indentation elastic modulus and the indentation hardness.

The same method of linear regression was used to calculate the correlations for each preservation process independently (Fig. 5). A Fisher-Z statistical test revealed no significant difference between the correlations, particularly between groups C and S (p_value_ = 0.27).

### 3.4 Trabeculae-scale study: Correlation between mechanical parameters and thickness of indented bone trabeculae

**Fig. 6:**
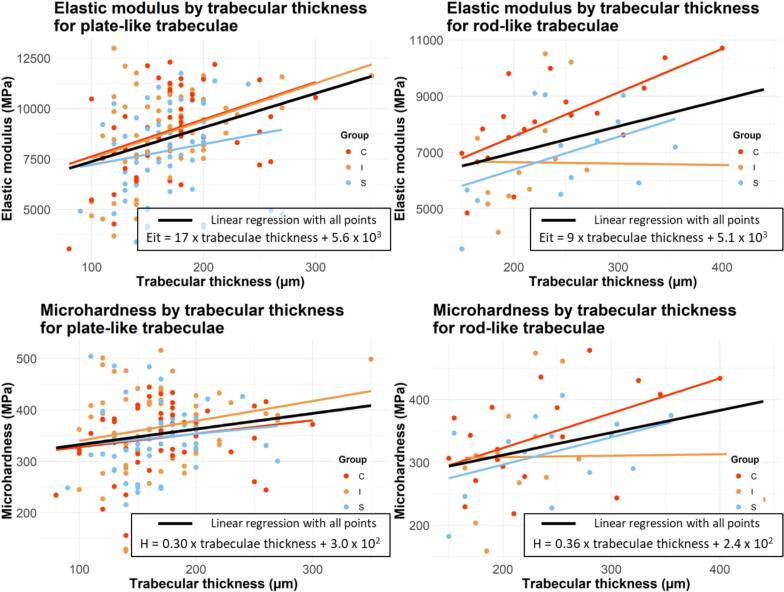
Effect of trabecular thickness of the indented trabeculae on mechanical properties for rod-like and plate-like trabeculae by preservation process with linear regression.

Each indented bone trabecula was analyzed with ImageJ and identified as a plate or rod using the ellipsoid factor (Doube, 2015). 22 % of the bone trabeculae were rods, and 78 % were plates. The indented trabeculae thickness was also measured. First, regarding the shape of the trabeculae, the plates' indentation elastic modulus and indentation hardness were significantly higher than those of the rods by 16.5 % and 8.9 %, respectively (p_value_ = 0.025 and p_value_ = 0.0003, respectively). Secondly, for both plates and rods, the indentation elastic modulus and indentation hardness increased significantly with the indented trabeculae thickness (Fig. 6). Linear regressions of indentation elastic modulus and hardness as a function of trabeculae thickness are shown in Fig. 6. The equations would be used to implement constitutive laws that depend on the microarchitecture of the patient's bone. Regarding the effect of the preservation process, while gamma irradiation has a significant effect on the correlation between indented trabeculae thickness and indented elastic modulus of rod-like trabeculae (Fisher-z test, p_value_ = 0.03), the supercritical CO_2_ process has no significant effect on the correlation between indented trabeculae thickness and mechanical properties for both plate and rod-like trabeculae (Fisher-z test, p_value_ > 0.05).

## 4 Discussion

The present work aimed to analyze the impact of a supercritical CO_2_ process on both the microarchitecture and the mechanical properties of the trabecular bone of the femoral head on a microscopic scale. We compared 3 preservation processes: freezing at −20 °C (Group C), gamma rays irradiation followed by freezing at −20 °C (Group I), and supercritical CO2 delipidation followed by chemical treatment and gamma irradiation (Group S).

### 4.1 Microarchitecture and mechanical properties on a microscale analysis

To the best of our knowledge, this is the first assessment of the impact of supercritical CO_2_ on the mechanical properties of bone at the microscopic scale using indentation methods.

First, the results concerning microarchitecture are consistent with existing studies (Porrelli et al., 2022; Saers et al., 2016; Krieger et al., 2025). Gamma irradiation and the supercritical CO_2_ process had no significant effect on the microarchitecture.

Secondly, concerning the mechanical properties, the mean values for indentation elastic modulus and indentation hardness were 8,27GPa and 347 MPa, respectively, which is consistent with existing studies on femoral heads in which the values are within [4–10 GPa] and [270 - 500Mpa], respectively (Soldati et al., 2022; Renault et al., 2020; Sihota et al., 2021; Ojanen et al., 2015). As demonstrated in Table 1, our present study shows that gamma irradiation and the supercritical CO_2_ processes have no significant effects on indentation elastic modulus and indentation hardness. On the contrary, some cleaning and sterilization processes without supercritical CO_2_ delipidation have been found to affect the mechanical properties measured with microindentation. Yin et al. indeed showed that enzymatic maceration significantly reduced the hardness of cortical bone (Yin et al., 2010). Interestingly, Sato et al. compared the hardness of several bone allografts and found that the properties of each graft varied according to the process used (Sato et al., 2012). Regarding gamma sterilization, two existing studies suggested that gamma irradiation increases hardness(Allaveisi et al., 2014; El-Hansi et al., 2020), but one study by Nikolaeva et al. suggested that irradiation slightly reduces hardness(Nikolaeva et al., 2023b). Our results show that gamma irradiation tends to induce a slight increase in hardness, which is in accordance with the two studies (Allaveisi et al., 2014; El-Hansi et al., 2020).

### 4.2 Correlation between bone volume fraction and the mechanical properties

The results show that indentation hardness and indentation elastic modulus increase with the bone volume fraction (BV/TV). This result is consistent with an existing study (Renault et al., 2020). Thus, the mechanical properties of trabecular bone appear to be initially dependent on the bone volume fraction and, subsequently, to a lesser extent, on the preservation process. It is, therefore, relevant to study the linear regression between indentation elastic modulus and bone volume fraction in order to estimate the effect of the supercritical CO_2_ process on the trabecular bone. For a bone volume fraction of 0.24 (the average bone volume fraction in our study), the indentation elastic modulus tends to be slightly reduced by 4 % due to irradiation and by 5 % due to the supercritical CO_2_ process (which also includes gamma irradiation). Thus, the reduction in indentation elastic modulus appears to be primarily caused by gamma irradiation, rather than by supercritical CO_2_.

Our correlation study suggests that the indentation elastic moduli of both groups I and S are lower than the indentation elastic modulus of group C when the bone volume fraction is lower (Fig. 4A). Existing studies in human adults have demonstrated that a lower bone volume fraction is associated with a higher collagen concentration (Ding et al., 2002). A negative effect of gamma irradiation on collagen fibers has already been observed (Singh et al., 2016; Shin et al., 2024; Sun et al., 2020). Since samples with a low BV/TV have a greater collagen concentration (compared to those with a high BV/TV), they are more likely to be altered by the gamma irradiation process. In other words, grafts with a low BV/TV would be more sensitive to collagen fiber damage induced by gamma irradiation.

### 4.3 Trabeculae-scale study: Correlation between mechanical parameters and thickness of indented bone trabeculae

As demonstrated in a previous study (Wojtkow et al., 2019), the mean trabecular thickness has been shown to be positively correlated with both the indentation elastic modulus and the indentation hardness. In this study, we investigated the correlation between mechanical properties and the trabecular thickness of each bone trabecula that has been indented. Firstly, the indentation elastic modulus and indentation hardness increased significantly with the thickness of the bone trabeculae for both plates and rods. This outcome confirms the result concerning the correlation between mean trabecular thickness and the mechanical properties, at both macro and microscopic scales (Wojtkow et al., 2019). On the other hand, the indentation elastic modulus and indentation hardness of the plates exhibited higher values than those of the rods (p_value_ = 0.025 and p_value_ = 0.0003, respectively), which is consistent with existing studies (Yu et al., 2021; Wang et al., 2015). However, the data presented in the 4 graphs of Fig. 5 show that the points are widely scattered, indicating that the thickness of the indented trabeculae is not the only factor influencing the mechanical properties at a microscopic scale. Other parameters (trabeculae orientation, connectivity, mechanical or chemical treatment) may also have a significant impact on the mechanical properties.

### 4.4 A comparison of the effect of the supercritical CO_2_ process at microscopic and macroscopic scales

This study on the effect of the supercritical CO_2_ process on the trabecular bone at the microscopic scale is complementary to an earlier study on the effect of the supercritical CO_2_ process on mechanical properties at the macroscopic scale (Krieger et al., 2025). The findings of the present study demonstrate that the process has no significant influence on the mechanical properties and microarchitecture of bone at these two scales. However, the non-significant effect on the elastic modulus was slightly more accentuated at the macroscopic scale, showing that the influence of this preservation process differs according to the investigation scale.

### 4.5 Limitations

The present study is subject to certain limitations. First, we had no information concerning the donors' medical history or the reasons for their arthroplasty. Nevertheless, the selection criteria for femoral head harvest corresponded to those used by tissue banks distributing bone allografts. Secondly, we used a small number of samples due to the difficulties of retrieving surgical wastes from hip arthroplasty. As a result, we observed some variability under the same conditions, reflecting the biological variability of donors (age, health, and/or bone quality). In future studies, with a greater number of samples, we could investigate in greater detail the various individual characteristics that may influence the response of bone allografts to preservation processes. Finally, the study does not show whether mechanical and microarchitectural properties remain stable following implantation in the patient. The impact of the conservation processes on bone degradation and rehabilitation remains to be explored.

## 5 Conclusion

In conclusion, the supercritical CO_2_ process exerts no significant effect on either the microarchitecture or the mechanical properties measured with microindentation. A study of the correlation between mechanical properties and bone volume fraction (BV/TV) shows that gamma irradiation appears to alter indentation elastic modulus. In contrast, the process comprising supercritical CO_2_ treatment, chemical treatment, and gamma irradiation does not alter the material more than gamma irradiation alone does.

## CRediT authorship contribution statement

**Théo Krieger:** Data curation, Investigation, Methodology, Software, Visualization, Writing – original draft, Writing – review & editing. **Virginie Taillebot:** Conceptualization, Methodology, Project administration, Supervision, Validation, Writing – review & editing. **Aurélien Maurel-Pantel:** Resources, Software. **Claire Camy:** Investigation, Writing – review & editing. **Grégoire Edorh:** Conceptualization, Funding acquisition, Resources. **Matthieu Ollivier:** Conceptualization, Funding acquisition, Writing – review & editing. **Martine Pithioux:** Conceptualization, Funding acquisition, Methodology, Project administration, Supervision, Validation, Writing – review & editing.

## Funding

This work was supported by BIOBank® (Lieusaint, France).

## Declaration of competing interest

The authors declare the following financial interests/personal relationships which may be considered as potential competing interests: Théo Krieger reports financial support was provided by Biobank (Lieusaint, France). Théo Krieger reports a relationship with Biobank (Lieusaint, France) that includes: employment. Matthieu Ollivier reports a relationship with Newclip Technics that includes: consulting or advisory. Matthieu Ollivier reports a relationship with Stryker Orthopaedics that includes: consulting or advisory. If there are other authors, they declare that they have no known competing financial interests or personal relationships that could have appeared to influence the work reported in this paper.

## Data Availability

Data will be made available on request.
